# Identification and seasonality of rhinovirus and respiratory syncytial virus in asthmatic children in tropical climate

**DOI:** 10.1042/BSR20200634

**Published:** 2020-09-24

**Authors:** Giselmo Pinheiro Lopes, Ítalo Patrick Souza Amorim, Bruna de Oliveira de Melo, Carlos Eduardo Campos Maramaldo, Maria Rosa Quaresma Bomfim, Lídio Gonçalves Lima Neto, Matheus Silva Alves, Fabrício Brito Silva, Paulo Vítor Soeiro-Pereira, Angela Falcai

**Affiliations:** 1 Postgraduate Program in Environment, São Luís, Maranhão, Brazil; 2 Postgraduate Program in Microbial Biology, CEUMA University, São Luís, Maranhão, Brazil; 3 Postgraduate Program in management of Health Programs and Services, CEUMA University, São Luís, Maranhão, Brazil; 4 Graduate Program, Ceuma University, São Luís, Maranhão, Brazil; 5 Department of Pathology, Federal University od Maranhão, São Luís, Maranhão, Brazil

**Keywords:** allergy, asthma, climate, respiratory infection, rhinovirus, seasonality

## Abstract

**Introduction:** Asthma is a disease that has been associated with the presence of different genetic and socio-environmental factors.

**Objective:** To identify and evaluate the seasonality of respiratory syncytial virus (RSV) and human rhinovirus (RV) in asthmatic children and adolescents in tropical climate, as well as to assess the socioeconomic and environmental factors involved.

**Methods:** The study was conducted in a referral hospital, where a total of 151 children were recruited with a respiratory infection. The International Study of Asthma and Allergies in Childhood (ISAAC) protocol and a questionnaire were applied, and a skin prick test was performed. The nasal swab was collected to detect RV and RSV through molecular assay. National Meteorological Institute (INMET) database was the source of climatic information.

**Results:** The socio-environmental characterization of asthmatic children showed the family history of allergy, disturbed sleep at night, dry cough, allergic rhinitis, individuals sensitized to at least one mite. We identified RV in 75% of children with asthma and 66.7% of RSV in children with asthma. There was an association between the presence of RV and the dry season whereas the presence of the RSV was associated with the rainy season. Contributing to these results, a negative correlation was observed between the RSV and the wind speed and the maximum temperature (T. Max) and a positive correlation with precipitation.

**Conclusions:** The results suggest a high prevalence of RV and RSV in asthmatic children and the seasonality of these viruses were present in different climatic periods. This has significant implications for understanding short- and long-term clinical complications in asthmatic patients.

## Introduction

Asthma is a chronic inflammatory disease, characterized by an abrupt hypersensitivity reaction mediated by Immunoglobulin E [1]. The prevalence of asthma has increased worldwide in developed countries, leading to numerous hospitalizations and a considerable rise in morbidity and mortality [2]. Approximately 300 million people worldwide are affected by asthma. In Brazil, asthma is the fourth cause of death caused by respiratory diseases, and according to data from the Pan American Health Organization, Brazil has more than 15 million people with asthma [3].

This pathology is the result of the interaction of genetic, immune, and environmental factors. Environmental exposure to allergens, irritants, and other specific factors leads to the development and maintenance of asthma symptoms. Different studies previously showed the association between viral infections, wheezing in infants, and exacerbation of asthma [4–6]. Infections with respiratory syncytial virus (RSV) and/or human rhinovirus (RV) are a fundamental cause in children’s and adolescents’ respiratory tract diseases and a major cause of bronchitis in children [1,7]. Some authors have already demonstrated that viral infections are capable of exacerbating asthma. As well as the exposure to RSV and RV serves as a primer for the development of this disease [8,9]. The mechanism by which RSV exacerbates asthma is associated with the T-cell response characterized mainly by Th2 cytokine production, the same response observed during asthma episodes.

Viral infections incidence suffers influence from seasonal factors such as precipitation, temperature, humidity, and wind speed, associated with the prevalence of respiratory diseases [10]. In countries of temperate zones, upper respiratory tract infections being more frequent in autumn and spring, rising during winter, following weather changes [11]. A study conducted in Germany suggests that humidity and temperature are associated with hospitalizations due to lower respiratory tract infections by the Influenza virus, RSV, and RV [12]. Another study carried out in the Colombia, showed that the occurrence of acute respiratory infection in children was associated with air temperature and relative humidity [13]. A study conducted in China, in 2016, showed that RV was the main viral pathogen in wheezing children, especially in the summer [14]. Other study carried out in China in 2020, showed the seasonality of RSV infection in hospitalized children and correlated with temperature [15].

However, few studies have reported the seasonal variation of respiratory virus in tropical countries. The relationship between presence of respiratory virus and development of asthma has been controversial. The present study aims to identify and evaluate the seasonality of RSV and human RV in asthmatic children and adolescents in tropical climate, as well as to assess the socioeconomic and environmental factors involved.

## Materials and methods

### Climatic characterization

In the state of Maranhão, Brazil, the climate presents two well-established periods, the rainy season (months from January to June) and the dry season (months from July to August), which were defined according to weather studies by Silva et al. (2019) [16]. The data of meteorological parameters, including maximum daily, average and minimum temperature (°C), wind speed (km/h), relative humidity (%) and precipitation [17] were obtained from the National Meteorological Institute (INMET) between April 2018 and March 2019, the same period as the systematic collection of samples from patients in the study.

#### Patients and ethical statements

##### Subjects

The study was performed at Dr. Odorico de Amaral Matos Children’s Hospital in the City of São Luís – MA, Brazil, from April 2018 to March 2019. A total of 151 children aged 2–12 years were included in the present study. The sample size was calculated using PASS 15® software, with the following parameters: prevalence 21.1% of children with respiratory infection in ambulatory [18], level of significance (α) of 5%, 80% test power, and tolerable error of 8%.

Upon hospital admission due to infection symptoms, patients were characterized as asthmatic and non-asthmatic, biological samples were collected, and then an immediate hypersensitivity test was performed according to the descriptions and protocols below. The clinical diagnosis of asthma was determinate by physicians from the hospital according to Global Initiative for Asthma (GINA) criteria: dyspnea, chronic cough; wheezing; chest tightness or chest discomfort, particularly at night or in the early hours of the morning; spontaneous improvement with or use of specific medications for asthma (e.g., bronchodilators, steroid anti-inflammatories). Besides, individuals who had three or more wheezing episodes within 6 months were considered to be wheezing. The clinical presentation of viral infection was characterized by watery nasal secretion, moderate cough, low hyperthermia, and wheezing [19].

##### Inclusion and exclusion criteria

The inclusion criteria considered were aged between 2 and 12 years (until the collection of biological material) and presenting respiratory infection. Children with pre-existing chronic lung disease, such as pneumonia, tuberculosis, and whooping cough, or under nebulizer therapy, were excluded.

#### Application of International Study of Asthma and Allergies in Childhood and complementary questionnaires

The International Study of Asthma and Allergies in Childhood (ISAAC) questionnaire was used to define asthma, rhinitis, and atopic eczema, with objective questions about the signs and symptoms of respiratory tract diseases [20]. A complementary questionnaire was applied to investigate the socio-environmental factors and family history of volunteers.

#### Immediate hypersensitivity skin test (prick test)

The skin prick test (Immunotech, FDA Allergenic Ltda, Rio de Janeiro, Brazil) was performed using extracts from domestic dust mite (*Dermatophagoides pteronyssinus, Dermatophagoides farinae*, and *Blomia tropicalis*), cat, dog, grass, egg and milk, buffered saline (negative control), and histamine (positive control). The test was performed on the front of the forearm following the manufacturer’s instructions. Skin prick test responses were considered positive if the allergen caused a wheal with a diameter of at least 3 mm, after 30 min.

#### Identification of viruses and quantification of the viral species by quantitative polymerase chain reaction

##### Viral samples

Respiratory samples were collected through a nasopharyngeal swab. After obtaining samples, those were disposed of in a 15-ml conical tube containing 2 ml of phosphate-buffered saline (PBS). Biological materials were centrifuged (3500 rpm for 10 min), and the supernatant was collected and stored at −80°C.

### Extraction of total RNA

The total RNA was obtained using a set of QIAamp Viral RNA Mini Kit® reagents (QIAGEN, GmbH, Germany), following the manufacturer’s instructions [21].

### Synthesis of the cDNA

The cDNA synthesis from the viral RNA extracted was performed using the Reverse Transcriptase Super Script™ II reagent set (Invitrogen, Gaithersburg, U.S.A.), following the instructions of the manufacturer’s material.

### Quantitative polymerase chain reaction

The identification of respiratory viruses was performed by quantitative polymerase chain reaction (qPCR) in real-time using the TaqMan® fluorescence probes system (Life Technologies, Foster City, CA, U.S.A.). Probes PROBE FAM-OS (CTGTGTATGTGGAGCCTTCGTGAAGCT) and oligonucleotides FORWARD (GGCAAATATGGAAACATACGTGAA) REVERSE (TCTTTTTCTAGGACATTGTAYTGAACAG) primers for cDNA amplification of the RSV, and probes PROBE FAM-MGBNFQ (TCCTTCCGGCYCCTGAATG) and oligonucleotides FORWARD1 (AGCCTGCGTGGCTGCCTG), FORWARD 2 (CCTGCGTGGCGGCCARC) and REVERSE (CCCAAAGTAGTYGGTCCCRTCC) primers for amplification of RV cDNA, synthesized as previously described [22]. The viral genetic material was amplified by qPCR using 200 nM of each primer oligonucleotide and 300 nM of the FAM-labeled probe (Life Technologies, Foster City, CA, U.S.A.). Other RT-PCR reagents were used in Master Mix solution [25 nM MgCl_2_, 10 mM dNTPs, Uracil N-glycosylase (UNG, AmpEraseR) 30 U, Amplitaq Gold 150 U enzyme and reaction buffer]. A final volume of 20 μl was used per reaction, and the assays were performed in duplicate. The automated ABI Prism 7500 Fast automation equipment (Life Technologies, Foster City, CA, U.S.A.) was used for the amplification assays.

### Statistical analysis

To compare the proportions of the classificatory variables, the chi independence square at the level of 5 and 10% (*P*<0.05 and *P*<0.10) was used. In some situations, the Yates’ correction was applied. The binary logistic regression model was also used to analyze the effect between categorical and independent variables; a reference category odds ratio (OR = 1) was established, considering risk factors greater than 1 and protective factors less than 1. First, a univariable regression was performed, and then the multivariable, considering a significance level of 10% (*P*<0.10).

Pearson correlation coefficient (r) was used to analyze the correlation between RSV and RV with mean precipitation, mean temperature, and wind speed. The level of significance accepted for analysis was 10% (*P*<0.1). The programs used to perform the analysis were PASS 15 (2017)®, GraphPad Prism 8®, and IBM SPSS Statistic 20®.

## Results

### Clinical and socio-environmental characteristics

The clinical and socio-environmental characteristics of the included population are shown in Table 1. A total of 69.9% of asthmatic children with infection presented a family history of allergic diseases, with allergic parents and/or siblings (Table 1, *P*<0.05). The presence of wheezing was a crucial clinical feature in 100% of asthmatic children, and 88.2% had disturbed sleep associated with wheezing (Table 1, *P*<0.05). Other symptoms, such as dry cough and allergic rhinitis, were also associated with asthma (Table 1, *P*<0.05). We observed that the majority of children with RSV and RV infection were between 1 and 5 years old (Supplementary Figure S1).

**Table 1:** Socio-environmental and clinical characteristics of asthmatic and non-asthmatic children with respiratory infection

	Children and adolescents with respiratory infections		
Variable	Non-asthmatic (*n*=58)	Asthmatic (*n*=93)	*P*-value
**Age (median)**	5.5	4.5	
**Gender**			0.17
**Boys**	31 (53.4)	61 (65.6)	
**Girls**	27 (46.6)	32 (34.4)	
**Mother’s education**			0.37
**Fundamental**	16 (27.6)	33 (25.5)	
**Middle/Upper**	42 (72.4)	60 (64.5)	
**Family income**			0.16
**Up to one salary**	46 (79.3)	82 (88.2)	
**Above one salary**	12 (20.7)	11 (11.8)	
**Breastfeeding up to 6 months**			0.18
**No**	6 (10.3)	4 (4.3)	
**Yes**	52 (89.7)	89 (95.7)	
**Divide the room**			0.15
**No**	3 (5.2)	1 (1.1)	
**Yes**	55 (94.8)	92 (98.9)	
**Domestic animal exposure**			0.60
**No**	15 (25.9)	21 (22.8)	
**Yes**	43 (74.1)	71 (77.2)	
**Exposure to secondhand smoke**			0.30
**No**	33 (56.9)	61 (65.6)	
**Yes**	25 (43.1)	32 (34.4)	
**Mold on the wall**			0.40
**No**	28 (48.3)	52 (55.9)	
**Yes**	30 (51.7)	41 (44.1)	
**Basic sanitation**			0.73
**No**	32 (55.2)	54 (58.1)	
**Yes**	26 (44.8)	39 (41.9)	
**Family history of the disease**			0.03*
**No**	28 (48.3)	28 (30.1)	
**Yes**	30 (51.7)	65 (69.9)	
**Presence of wheezing**			0.001*
**No**	30 (51.7)	0 (0.00)	
**Yes**	28 (48.3)	93 (100)	
**Sleep disturbed at night**			0.01*
**No**	44 (75.9)	11 (11.8)	
**Yes**	14 (24.1)	82 (88.2)	
**Dry cough**			0.01*
**No**	37 (63.8)	31 (33.3)	
**Yes**	21 (36.2)	62 (66.7)	
**Rhinitis**			0.03*
**No**	36 (62.1)	40 (43)	
**Yes**	22 (37.9)	53 (57)	
**Eczema**			0.06
**No**	36 (62.1)	71 (76.3)	
**Yes**	22 (37.9)	22 (23.7)	

The number in parenthesis represents the percentage value relative to the study group. *Chi-square test—Fisher’s test. *P*<0.05.

### Hypersensitivity test

The hypersensitivity test showed that 78.9% of asthmatic children with infection were sensitized to at least one allergen (Table 2, *P*<0.05); 61% were sensitized to Derp, 51.9% to Derf, 37.7% to Blot, 88.5% to Pera, 76.6% to cat and 23.6% to dog (Table 2, *P*<0.05).

**Table 2:** Allergic sensitization of the asthmatic and non-asthmatic children with respiratory infection

	Children and adolescents with respiratory infections		
	Non-asthmatic (*n*=58)	Asthmatic (*n*=93)	*P*-value
**Sensitized by at least one allergen**			
**No**	27 (57.4)	16 (21.1)	0.01*
**Yes**	20 (42.6)	60 (78.9)	
***Dermatophagoides pteronyssinus***			
**No**	33 (68.8)	30 (39)	0.002*
**Yes**	15 (31.3)	47 (61)	
***Dermatophagoides farinae***			
**No**	37 (77.1)	37 (48.1)	0.001*
**Yes**	11 (22.9)	40 (51.9)	
***Blomia tropicalis***			
**No**	41 (85.4)	48 (62.3)	0.008*
**Yes**	7 (14.6)	29 (37.7)	
***Periplaneta americana***			
**No**	48 (100)	68 (88.3)	0.01*
**Yes**	0 (0.00)	9 (11.7)	
***Blatella germanica***			
**No**	46 (95.8)	66 (85.7)	0.12
**Yes**	2 (4.2)	11 (14.3)	
**Cat**			
**No**	44 (91.7)	57 (74)	0.01*
**Yes**	4 (8.3)	20 (26)	
**Dog**			
**No**	44 (93.6)	59 (76.6)	0.01*
**Yes**	3 (6.4)	18 (23.4)	
**Gramineae**			
**No**	47 (100)	74 (96.1)	0.28
**Yes**	0 (0.00)	3 (3.9)	
**Egg**			
**No**	46 (95.8)	76 (98.7)	0.55
**Yes**	2 (4.2)	1 (1.3)	
**Milk**			
**No**	45 (93.8)	77 (100)	0.05
**Yes**	3 (6.3)	0 (0.00)	

The number in parenthesis represents the percentage value relative to the study group. *Chi-square test—Fisher’s test. *P*<0.05.

### Identification and seasonality of RV and RSV in asthmatic or non-asthmatic children

It was observed that 75% of RV-infected children were asthmatic patients, which resulted in a statistically significant difference. The RV was associated with the dry climate period of the region (Table 3, *P*<0.10). While 66.7% of RSV-infected children were non-asthmatic, this did not present a statistical difference between the asthmatic and non-asthmatic groups (Table 3, *P*>0.10). When analyzing the seasonality of RSV, an association was observed between the presence of the virus and the rainy season (Table 3, *P*<0.10).

**Table 3:** Identification and seasonality of RV and RSV in children and adolescents with and without asthma

	Children and adolescents with respiratory infections					
	RV		*P*-value	RSV		*P*-value
	Absent (*n*=115)	Present (*n*=36)		Absent (*n*=124)	Present (*n*=27)	
**Non-asthmatic**	49 (42, 6)	9 (25)	0.07*	49 (39, 5)	9 (33, 3)	0.66
**Asthmatic**	66 (57, 4)	27 (75)		75 (60, 5)	18 (66, 7)	
**Rainy climate**	80 (69, 6)	7 (19, 4)	0.001^†^	67 (54)	20 (74, 1)	0.08*
**Dry climate**	35 (30, 4)	29 (80, 6)		57 (46)	7 (25, 9)	

The number in parenthesis represents the percentage value relative to the study group. *Chi-square test—Fisher’s test. *P<0.10. ^†^*P*<0.05.

Asthma was associated with family history variables, disturbed sleep associated with wheezing, dry cough, allergic rhinitis, children sensitized to at least one mite, and sensitization to allergens: Derp, Derf, and Blot. All variables were considered risk factors (Table 4, *P*<0.05). Asthma and dry climate were associated, as risk factors, to RV infection, while the dry period was a protective factor against the RSV (Table 4, *P*<0.05).

**Table 4:** Logistic regression analysis of principal predictors of asthma, climate and RV or RSV infection

	*P*-value	OR (95% CI)
**Asthma**		
**Family history of asthma**	0.002*	2.16 (1.09–4.27)
**Sleep disturbed at night**	0.001*	23.4 (9.8–55.90)
**Dry cough**	0.002*	3.13 (1.54–6.30)
**Rhinitis**	0.002*	2.16 (1.10–4.24)
**Sensitized by at least one allergen**	0.001*	5.06 (2.2–11.20)
***Dermatophagoides pteronyssinus***	0.001*	3.44 (1.60–0.73)
***Dermatophagoides fariane***	0.002*	3.63 (1.62–8.15)
***Blomia tropicalis***	0.007*	3.5 (1.40–8.90)
** RV**		
**Asthma**	0.060^†^	2.22 (0.96–5.10)
**Dry climate**	0.001*	9.46 (3.78–23.6)
**Rainy climate**	0.001*	0.10 (0.42–0.26)
** RSV**		
**Asthma**	0.595	1.27
**Dry climate**	0.060^†^	0.41 (0.16–1.04)
**Rainy climate**	0.060^†^	2.43 (0.95–6.13)

Logistic regression analysis. *P<0.05 ^ †^*P*<0.1.

### Correlation between the presence of RSV and RV and the climatic variables

Since we observed a difference in the seasonality of the virus, it was evaluated the climatic variables as wind speed, precipitation, maximum temperature (T. Max) and minimum temperature (T. Min). The data show a positive correlation with the precipitation variable (Table 5, *P*<0.10).

**Table 5:** Correlation between the presence of RSV and RV and the climatic variables of wind speed, precipitation, T. Max, and T. Min

	RSV	RV	Wind velocity	Precipitation	T. Max
**RV**	0.15				
	0.62				
**Wind velocity**	−0.72	0.3			
	0.01^†^	0.33			
**Precipitation**	0.51	−0.23	−0.86		
	0.09^†^	0.45	0.0006*		
**T. Max**	−0.78	0.22	0.9	−0.83	
	0.003*	0.47	0.0001*	0.001*	
**T. Min**	−0.17	0.42	0.42	−0.28	0.37
	0.59	0.17	0.16	0.36	0.23

Abbreviation: T, temperature. Spearman’s rank-order correlation. *P<0.05 ^ †^*P*<0.1.

**Figure 1:**
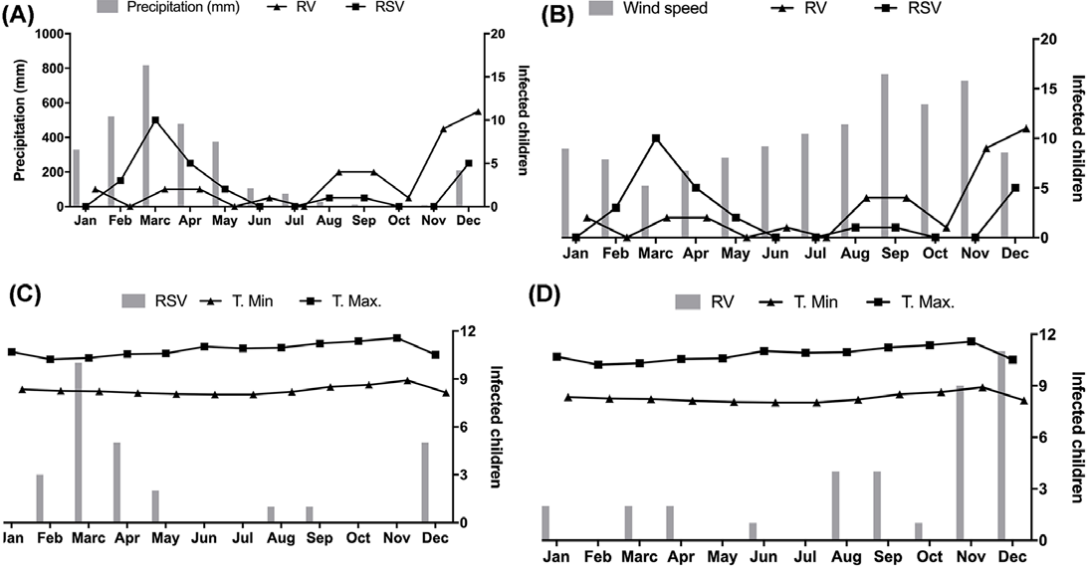
Time analysis between the climatic variables and the presence of the virus (**A**) Precipitation. (**B**) Wind speed. (**C**) Maximum and minimum temperatures associated with RSV. (**D**) Maximum and minimum temperatures associated with HRV.

Also, we performed a temporal analysis of the data. Figure 1A shows the peak of RSV and RV infections. In the case of RSV infection, the peak occurs between February and March in the months with the highest precipitation. The peak of RV infection occurred between November and December, at the end of the dry period. These observations show the influence of climatic variations on the distribution of these infections. In the temporal analysis of the wind velocity, it is observed that the peak incidence of the RSV is in March, which is associated with low wind speed (Figure 1B). The time series of T. Max and T. Min and the RSV is shown in Figure 1C, and the RV is shown in Figure 1D.

## Discussion

RSV and human RV infections are important factors in diseases affecting the respiratory tract in children. This research showed that respiratory viral infections in childhood were associated with asthma triggering or development, and with essential predictor factors, like a family history of allergy, wheezing, and sensitization to allergens. These data were crucial to show the high prevalence of these viruses in children and adolescents with asthma and also necessary to show an association between the presence of RV and asthma. Also, the seasonality of the RV and RSV were observed concerning the rainy and dry climate and a correlation with the variables of precipitation, wind speed, and maximum temperature.

Our results confirm the known relationship between a family history of allergies. The chances of the children becoming asthmatic is 50% when one of the parents and/or sibling presents atopy [23]. Our data confirmed that symptoms such as wheezing, disturbed sleep at night, cough and asthma, and at least once weekly seizures and rhinitis were risk factors for asthma. The previous study on the prevalence of asthma and associated in the city of São Luís do Maranhão, demonstrated (through ISAAC and supplementary questionnaires) that approximately 15% of the 13–14 students studied presented asthmatic symptoms, and the most associated factors were family history of asthma, rhinitis allergic, and wheezing at the onset of life [24].

In the first 3 months of life, exposure to allergens, as mite and cockroach, has been associated with the development of recurrent wheezing and asthma in children [25,26]. Our results showed that patients with infection and asthma were associated with sensitized by *D. pteronyssinus, D. farinae, B. tropicalis, P. americana*, and cat and dog allergens. All sensitized subjects, at least to one allergen, presented asthma. In the city of São Paulo, *D. pteronyssinus and B. tropicalis* account for 50 and 26% of the allergens present in house dust, respectively [27]. In Surakarta, Indonesia, the sensitization of house dust allergens was found to be higher for *D. farinae* (62.1%), followed by *D. pteronyssinus* (51.7%) and *B. tropicalis* (48.3%). The highest was *D. pteronyssinus* sensitization in recurrent asthma [28].

As crucial as sensitization to allergens, bronchitis induced by these viruses during early childhood is strongly linked to the later development of allergies and asthma [8]. Evidence suggests that genetic and environmental factors determine the type of immune response in RSV and RV infections, which contributes to the development of asthma [29]. We observed an association of asthma with RV. Other authors have demonstrated that 92.2% of the cases of exacerbation of asthma were manifested by the presence of viral agents with emphasis on respiratory infections caused by human RV and that the period of viral circulation may be correlated with the frequency of cases of asthma exacerbation [30].

Regarding the presence and seasonality of RV and RSV, our data showed an association between RV and dry climate, and an association between RSV and rainy climate.

Regarding the presence and seasonality of RV and RSV, our data showed an association between RV and dry climate, and an association between RSV and rainy climate. In Maranhão, the equatorial climate is dominant in the western part of the state, providing rainfall and high temperatures; the rest of the region is influenced by the tropical climate, with higher rainfall rates in the first months of the year, promoting respiratory viral infections. Once this difference between the rainy and dry climate was observed, a correlation was made with the presence of viruses and climatic variables (precipitation, wind speed, T. Max and T. Min). There was a positive correlation between RSV infection with precipitation and a negative correlation with T. Max and wind speed. These results corroborate a study conducted in Western Australia, which observed an incidence of RSV, and the climate of this region is characterized by the rainy season. The authors suggest that climatic variables of precipitation and humidity may favor viral survival [31].

A study showed that the time and seasonal permanence of RSV were consistent in countries from year to year, the associations between RSV and climate varied in years and geographic locations. The RSV reached a peak in climates with high annual rainfall (Bangladesh, Guatemala, and Thailand) during the humid months, peaking during the colder months in moderately hot (China) and arid (Egypt) regions [1]. Besides, Paynter et al., 2015 also demonstrated that the seasonality of the acute RSV respiratory infection rate in children in hospital sectors in the Philippines was related to higher precipitation [32]. These RSV prevalence variations, driven by a range of environmental factors, which may overcome and increase the deleterious effect of asthma inflammation.

## Conclusions

The incidence of RSV and RV respiratory infections varies according to seasonal climatic differences, which may be related to the pathogen survival and host susceptibility. Besides, there was an association of RV infection with asthma. Understanding the factors involved in the transmission of respiratory viruses may help predict future outbreaks and development of interventions. That information can represent an innovative approach to manage asthma in a prophylactic way.

## Perspectives

- The prevalence of asthma has increased worldwide in developed countries, leading to numerous hospitalizations. The association between viral infections, wheezing in infants, and exacerbation of asthma is well established. Viruses are known to have different seasonality in each region. This is the first study that identifies and observes the seasonality of RV and RSV in a tropical country and associates this seasonality with the development of asthma.
- Our results show a high prevalence of RV and RSV and an association of RV with asthma. In addition, a correlation was observed between the dry season and the presence of RV and the rainy season with the presence of the RSV.
- These data contribute to define public health policies and management and intervention strategies for the control of asthma and medium-term improvement in the quality of life of patients with allergic manifestations.

## Supplementary Material

Supplementary Figure S1

## Abbreviations

ISAAC: International Study of Asthma and Allergies in Childhood
qPCR: quantitative polymerase chain reaction
RSV: respiratory syncytial virus
RV: rhinovirus
T. Max: maximum temperature
T. Min: minimum temperature
