# Supplementary figures and images for: Identification and seasonality of rhinovirus and respiratory syncytial virus in asthmatic children in tropical climate
Source: Biosci Rep. 2020 Sep 24;40(9):BSR20200634. doi: 10.1042/BSR20200634 (PMC7517263; doi:10.1042/BSR20200634)

**Supplementary figure.** Relationship between children with RV and RSV infections and age.

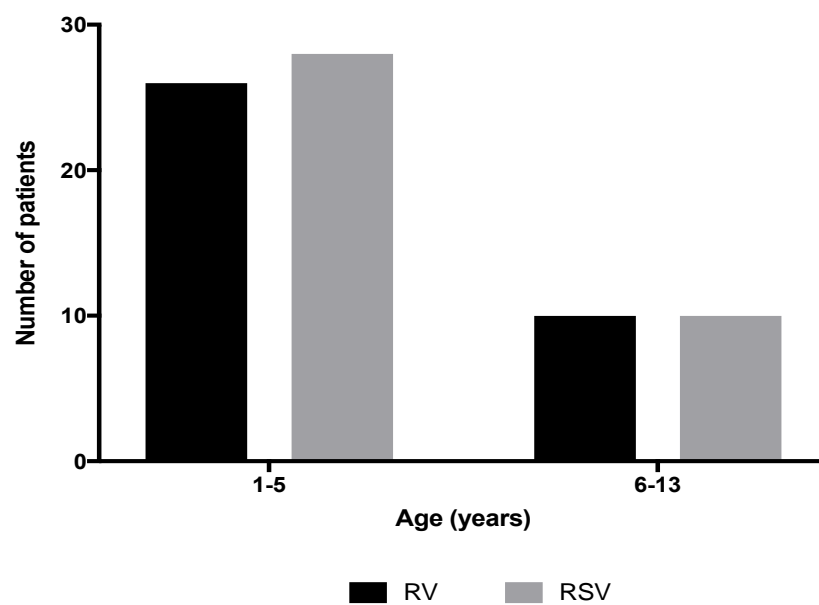

Supplement: Supplementary Figure S1 [file BSR-2020-0634_supp.pdf]
